# Metacognitive Information Theory

**DOI:** 10.1162/opmi_a_00091

**Published:** 2023-07-21

**Authors:** Peter Dayan

**Affiliations:** Max Planck Institute for Biological Cybernetics, Tübingen, Germany; University of Tübingen, Tübingen, Germany

**Keywords:** metacognition, information theory, signal detection theory, scoring rules, m-ratio

## Abstract

The capacity that subjects have to rate confidence in their choices is a form of metacognition, and can be assessed according to bias, sensitivity and efficiency. Rich networks of domain-specific and domain-general regions of the brain are involved in the rating, and are associated with its quality and its use for regulating the processes of thinking and acting. Sensitivity and efficiency are often measured by quantities called meta–*d*′ and the M-ratio that are based on reverse engineering the potential accuracy of the original, primary, choice that is implied by the quality of the confidence judgements. Here, we advocate a straightforward measure of sensitivity, called meta–𝓘, which assesses the mutual information between the accuracy of the subject’s choices and the confidence reports, and two normalized versions of this measure that quantify efficiency in different regimes. Unlike most other measures, meta–𝓘-based quantities increase with the number of correctly assessed bins with which confidence is reported. We illustrate meta–𝓘 on data from a perceptual decision-making task, and via a simple form of simulated second-order metacognitive observer.

## INTRODUCTION

The confidence that we apportion to our recollections, cognitions, decisions and actions can play a critical role in the preparations we make for success or failure; in determining whether we need to collect more external information or more samples of internal information before committing ourselves; in regulating the learning that we should do when outcomes are, or are not, as expected; and in communicating with others, for instance when engaging in collective decision-making (Bahrami et al., 2010; De Martino et al., 2013; Fleming, 2021; Fleming et al., 2012; Kepecs & Mainen, 2012; Nelson & Narens, 1990; Schulz et al., 2020). Confidence, as one of the simplest forms of higher-order or self-reflective assessment about one’s own cognitive processes, has also been (sometimes controversially) influential in modern theories of awareness (Fernandez-Duque et al., 2000; Lau, 2022; Lau & Rosenthal, 2011). Furthermore, various impairments of metacognition are central in a number of psychiatric conditions, for instance, with possibly exorbitant requirements for confidence helping underpin excessive checking in forms of obsessive compulsive disorder; or substantial over-confidence helping reinforce the persistent apparently erroneous conclusions drawn by those suffering from delusional disorders (Hoven et al., 2019; Rouault et al., 2018; Seow et al., 2021; Sun et al., 2017). Various regions of the prefrontal cortex, anterior cingulate cortex, insular cortex and the precuneus have been implicated in making such judgements and using them to control our cognition (for a meta-analysis of a wealth of studies, see Vaccaro & Fleming, 2018).

It has therefore long been recognized that is critical to measure the nature and quality of confidence judgements. At stake are three, related quantities: *bias*, *sensitivity*, and *efficiency* (Fleming & Lau, 2014; Galvin et al., 2003; Maniscalco & Lau, 2012; Nelson, 1984). For concreteness, consider a simple perceptual decision-making problem: judging whether a Gabor patch is tilted left (L) or right (R) of vertical. The sensory input *α* can abstractly be regarded as a noisy version of *d* = −1 (L) or *d* = 1 (R). On each trial, subjects report their decision (the ‘action’ *a*) about the tilt—this is often called a type 1 judgement—and also their degree of confidence in the rectitude or accuracy of that decision (the ‘rating’)—a type 2 judgement. For convenience, we consider the original report as coming from an ‘actor’, and the confidence judgement from a ‘rater’; although these are, of course, the same individual (Schulz et al., 2023). The type 1 judgement is the topic of conventional signal detection theory (Green & Swets, 1966), with accuracy being quantified by such measures as type 1 sensitivity or *d*′. If the rating is interpreted as just the probability that the type 1 decision is correct (Pouget et al., 2016), then metacognitive bias measures the overall calibration of the rating—whether subjects tend to think that they are more or less accurate than they actually are. Of course, a subject could be metacognitively unbiased if she reported the correct overall probability of being correct on every trial, independent of the actual observation (like a well-calibrated, but useless, weather forecaster reporting on every day of the year, the overall mean probability of rain; Dawid, 1982). Thus, metacognitive sensitivity measures the adaptability of the rating to the actual rectitude on a trial-by-trial basis—an ideal rater would have perfectly predictive error monitoring, rating correctly on a trial-by-trial basis whether the type 1 decision is going to be proved correct or incorrect. However, metacognitive sensitivity is not the whole story—the rater has a particularly easy job if the type 1 action is generally correct—it would be hard for the rater to be incorrect. Thus metacognitive efficiency attempts to correct the sensitivity for the quality of inference. Of course, metacognitive bias also has an impact: a thoroughly metacognitively biased rater who declares themselves fully confident on every trial, even when she in fact errs, would necessarily be fully insensitive and inefficient.

It might seem obvious, at least to the Bayesian decision theorists amongst us, that sensible observers would use all the information available to make their type 1 choice on a trial (*a* = 1 if *P*(*d* = 1|*α*) > 0.5), and the same information to make their type 2 rating (*P*(*d* = 1|*α*)) about their type 1 choice. This would be bias-free, and would leave sensitivity and efficiency at maximal values given the decision-maker’s perceptual capacities (*d*′). This would render nugatory the metacognitive measures. However, empirical findings do not accord with this expectation (for instance, it is impossible for the rating to be of a less than 50% chance of being correct; whereas subjects can actually be aware of upcoming errors before they occur (Gehring et al., 1993); also evident in signals that likely emanate from the anterior cingulate cortex (Botvinick et al., 2004; Carter et al., 1998; Dehaene et al., 1994; Kerns et al., 2004). Thus, there are various accounts in which, for instance, in a so-called second order model, the internal rater has access to both additional information after the type 1 decision has been made (for instance from so-called post-decisional information, which we later call *γ*, that has not been processed at the time that the decision is registered), and/or only a noisy internal report (*β*) of the information *α* that the actor used in making the type 1 decision in the first place (Fleming & Daw, 2017; Jang et al., 2012). The rater could also suffer from noise in their metacognitive judgement or report (Guggenmos, 2022, Shekhar & Rahnev, 2021). In cases such as this, it is possible to have metacognitive hypo- or hyper-sensitivity, and for the rater to predict errors.

When the type 2 decision is not a trivial function of type 1 information, much effort has gone into determining useful measures of metacognitive sensitivity and efficiency (Evans & Azzopardi, 2007; Ferrell & McGoey, 1980; Fleming & Lau, 2014; Galvin et al., 2003; Guggenmos, 2021; Kunimoto et al., 2001; Maniscalco & Lau, 2012; Nelson, 1984). One influential and attractive idea has been to imagine the type 1 decisions that the rater would have been able to make, and assess the notional type 1 sensitivity of this rater (Maniscalco & Lau, 2012, 2014). This value is called meta–*d*′, and has the attractive characteristic of being directly comparable to the actual type 1 sensitivity. Meta–*d*′ can be assessed by fitting the actual confidence statistics to the confidence statistics that would have been predicted by the imaginary type 1 choices of the rater. It is then possible to create a metacognitive efficiency measure that adjusts for the underlying ease of the decision-making problem by comparing meta–*d*′ to *d*′ either subtractively (meta–*d*′ — *d*′) or divisively (the so-called M-ratio, which is meta–*d*′/*d*′).

Meta–*d*′ and the M-ratio are widely used as measures of meta-cognitive effectiveness (Barrett et al., 2013). However, along with some obvious assumptions (such as that ratings are monotonic in expected accuracy), they have some less desirable characteristics, including remaining dependency of the M-ratio on type 1 performance (Guggenmos, 2021, at least some aspects of which are, as noted above, inevitable), and on metacognitive bias (Xue et al., 2021, which is arguably less so). Here, along with the common observation that there is no reason to expect subjects’ empirical confidence judgements to fit an assumed type 1 decision-process exactly, which means that the assessment of metacognitive efficiency could be inaccurate, we focus on the fact that the M-ratio does not take explicit account of the number of levels of confidence rating that subjects might be able to provide. A rater who can make a fine discrimination between being correct within the intervals [80, 85)% or [85, 90)% might reasonably demand to be considered more sensitive (and more efficient) than one with a single rating ‘bucket’ for the whole range [80, 90)%. Given a rater whose confidence is perfectly consistent with a type 1 decision, this excess discriminability will normally have no benefit from the perspective of meta–*d*′.

Here, we introduce and explore a natural alternative to meta–*d*′ and the M-ratio, namely meta–𝓘 and two forms of a meta–𝓘-ratio (called meta–${𝓘}_{1}^{r}$ and meta–${𝓘}_{2}^{r}$), which are based on the mutual information between the rectitude of the actor and the confidence ratings. The mutual information is straightforward to compute for conventional rating buckets, makes fewer assumptions about the ratings, other than that they are distinct and, ideally, suitably predictive of differences in accuracy, and increases naturally with the granularity of the ratings. The mutual information is related to measures that are based on the correlation between accuracy and confidence (Nelson, 1984), although it is, for instance, completely agnostic to any bias. We first illustrate meta–𝓘-based measures on confidence data from Shekhar and Rahnev (2021). Then, to examine their properties in detail, we use a simple realization of a second-order rater (Fleming & Daw, 2017; Guggenmos, 2022; Jang et al., 2012; Mamassian & de Gardelle, 2022; Schulz et al., 2023), for which we can precisely unpick the nature of metacognitive sensitivity.

## META–𝓘

Consider a simple perceptual decision-making task such as that reported in Shekhar and Rahnev (2021). Here, on each of 2800 trials *t*, participants saw for just 100 ms a noisy Gabor patch (of one of three different contrasts, defining three *conditions*) that was tilted either to the left or right of vertical (we write this as *d*^*t*^ = ±1), and used a single scale to report the direction of the tilt (*a*^*t*^ = ±1) and a continuous confidence rating (*c*^*t*^) about the accuracy of their choices (whose true value is *r*^*t*^ = *d*^*t*^ × *a*^*t*^). Confidence reporting in this experiment restricted *c*^*t*^ to being between 0.5 and 1.

**Figure 1.**
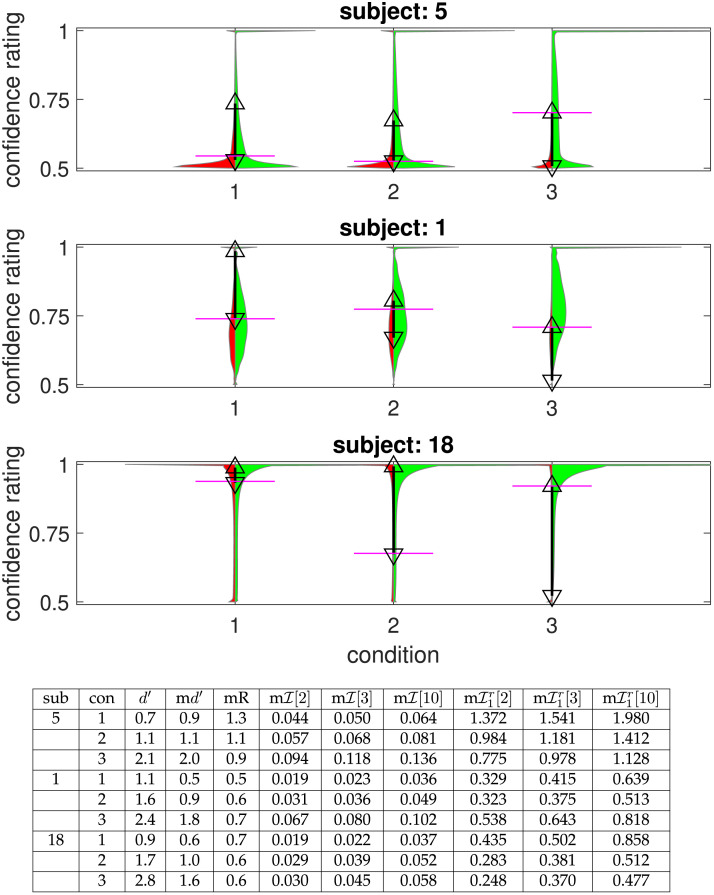
**Confidence and accuracy for three selected subjects (‘sub’).** Each plot shows the distribution of confidence reports for incorrect (red) and correct (green) choices for each of the three contrast conditions (‘con’). The total area of the violin plots is normalized, so the overall accuracy is evident in the sizes of the green areas. The magenta bars show the division of confidence ratings into two bins that approximately maximize meta–𝓘; the lower and upper triangles show the same for three bins. The table provides the numerical values of the various sensitivity and efficiency measures for these subjects. m*d*′ is meta–*d*′, mR is the M-ratio, m𝓘[*n*] is meta–𝓘 for *n*, approximately optimally-positioned, confidence bins, m${𝓘}_{1}^{r}$[*n*] is meta–${𝓘}_{1}^{r}$ for those same bins. Data from Shekhar and Rahnev (2021).

To illustrate the issues for measuring the quality of metacognition, Figure 1 shows violin plots of the distributions of confidence reports for three selected subjects for incorrect (red) and correct (green) choices and for the three contrast conditions (1 is hardest; 3 is easiest). Subject 5 is biased to report low confidence; subject 18 to report high confidence; subject 1 is in the middle. We can see the reduction in incorrect responses with higher contrast (i.e., higher condition number); but also additional facets such as the spikes of very high confidence reports. Since confidence should provide information about accuracy, measures of meta-cognitive sensitivity report how closely related are *c*^*t*^ and *r*^*t*^. In terms of the plots in Figure 1, we would seek the mass of the green distributions to be higher than those of the red ones, with higher confidence ratings when the answer is actually correct.

As mentioned above, there is a wide variety of such measures, some of which are based on process models of the way that participants make decisions and rate confidence (e.g., Desender et al., 2021; Maniscalco & Lau, 2012; Shekhar & Rahnev, 2021), whereas others are agnostic to the process by which confidence judgements are made, and depend on some form of correlation between accuracy and confidence (Nelson, 1984). In both cases, raw assessments are influenced by the absolute accuracy, since, for instance, if the decision-making task is very easy, there is little uncertainty to which confidence could be sensitive. The table below the figure indicates *d*′, meta–*d*′ (written as m*d*′) and the M-ratio (mR). Here, these quantities were calculated using maximum likelihood fitting routines from Maniscalco and Lau (2012, 2014), in which the parameters of a naïve first order Bayesian rater are fit so that the distribution of its confidence ratings match those of each subject. From the M-ratio, we can see that, in this case, the order of the efficiency of these subjects is opposite to the order of their metacognitive bias.

**Figure 2.**
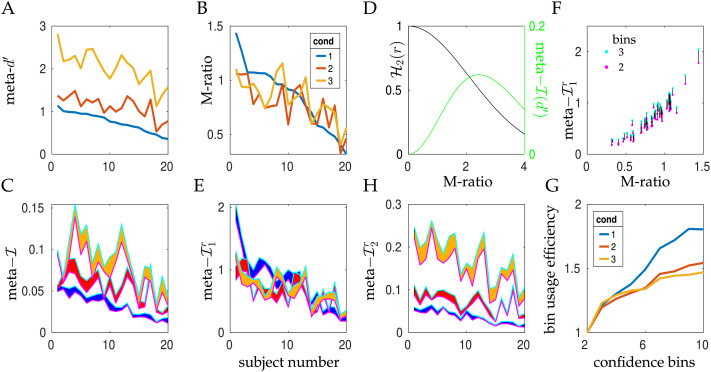
**Metacognition in Shekhar and Rahnev (2021).** (A) meta–*d*′ across the 20 subjects for the three contrast conditions (in order of contrast, blue, red, yellow), with the subjects sorted by the lowest contrast. (B) The M-ratio for the subjects, again sorted by the value in the lowest contrast condition. (C) meta–𝓘 for the subjects in the same sort order as in (A) for two (lower envelope in magenta) or three (upper envelope in green) confidence bins with optimized thresholds. The lozenges fill the areas between lower and upper envelope in the colours for the condition. (D) The two normalizers for assessing efficiency: meta–𝓘(*d*′) (green; right axis, used to define meta–${𝓘}_{1}^{r}$) and 𝓗_2_(*r*) (black; left axis, used to define meta–${𝓘}_{2}^{r}$) as a function of the actor’s *d*′. (E) meta–${𝓘}_{1}^{r}$ for the subjects in the same sort order as in (B) with the same plotting conventions as in (C). (F) The relationship between the M-ratio and meta–${𝓘}_{1}^{r}$ for the 60 combinations of subjects and conditions, for two (magenta) and three (green) confidence bins, joined by thin black lines for clarity. (G) The average across subjects of the ratio between meta–𝓘 (or equivalently meta–${𝓘}_{1}^{r}$ or meta–${𝓘}_{2}^{r}$) for 2 … 10 confidence bins to meta–𝓘 for just 2 confidence bins, for the three conditions. This shows that the subjects are, on average, able to use multiple bins to some good effect. (H) meta–${𝓘}_{2}^{r}$ for the subjects in the same sort order as in (B) with the same plotting conventions as in (C).

Figure 2A and B show meta–*d*′ and the M-ratio for all the subjects in Shekhar and Rahnev (2021), in the three contrast condition. The subjects are sorted differently in each figure in decreasing order of the sensitivity (Figure 2A) or efficiency (Figure 2B) for the most difficult condition (blue). We see that both measures tend to decrease together for all the contrast conditions, confirming past observations that there something generalizable about sensitivity and efficiency, at least for such closely related problems. Figure 2A also shows the dependence of meta–*d*′ on *d*′: as noted, these values are distinctly greater for the higher contrast conditions. Figure 2B shows that this characteristic is largely abolished for the M-ratio, in which the rater’s meta–*d*′ is normalized by the actor’s *d*′.

The measure meta–*d*′ (along with others mentioned above) is a model-based measure of sensitivity, in that one imagines that the confidence reports are the first order judgements for an actor with some particular, parametrized characteristics. By contrast, meta–𝓘 is a model-agnostic measure of metacognitive sensitivity which quantifies the mutual information between *r*^*t*^ and *c*^*t*^. Take the case that confidence is discrete (in Shekhar and Rahnev (2021), it is measured in 1/1000^ths^) and that we had been able to measure the full joint distribution of rating and confidence, *P*(*r*, *c*), with *P*(*r*) = ∑_*c*_ *P*(*r*, *c*); *P*(*c*) = ∑_*r*_ *P*(*r*, *c*). Then, the mutual information is the difference between two entropies (measured, for convenience, in bits). One entropy, which we write as 𝓗_2_(*r*), is the overall uncertainty about the accuracy of the actor. For binary choice, this quantity varies between 0 bits, if the actor is perfectly accurate, as *d*′ → ∞ (or indeed if the actor is perfectly inaccurate; always getting the answer wrong) and 1 bit, if the action *a* is completely uncorrelated with the truth *d*, which happens as *d*′ → 0. The second entropy, 𝓗_2_(*r*|*c*) is the weighted average uncertainty about the accuracy that remains after observing the confidence rating *c*, where the weights come from the probability of seeing that rating *c*. The confidence judgement is very sensitive and efficient if most of the initial uncertainty about the accuracy is removed by the rating, making this last term near 0.

More formally,

$$\text{meta}–𝓘={𝓗}_{2}\left(r\right)-{𝓗}_{2}\left(r|c\right)\;\text{where}$$ (1)

$${𝓗}_{2}\left(r\right)=h_{2}\left[P\left(r\right)\right]\;\text{is}\;\text{the}\;\text{entropy}\;\text{of}\;\text{the}\;\text{accuracy},$$ (2)

$${𝓗}_{2}\left(r|c\right)=\sum_{c}P\left(c\right)h_{2}\left[P\left(r|c\right)\right]\;\text{is}\;\text{the}\;\text{conditional}\;\text{entropy}\;\text{of}\;\text{accuracy}\;\text{given}\;\text{confidence};$$ (3)

$$h_{2}\left[P\left(x\right)\right]=\sum_{x=0;1}-P\left(x\right)\;\log_{2}\;P\left(x\right)\;\text{is}\;\text{the}\;\text{entropy}\;\text{of}\;\text{a}\;\text{Bernoulli}\;\text{random}\;\text{variable}$$ (4)

Mutual information is symmetric, so one can also write meta–𝓘 = 𝓗_2_(*c*) − 𝓗_2_(*c*|*r*). As is sometimes common, one could condition all these quantities on the action, and so report response-specific meta–𝓘(*a* = 1) and meta–𝓘(*a* = −1).

Table 1 provides an illustration of the way that one can calculate mutual information. Here, we show the four combinations of correct (*r* = 1) and incorrect (*r* = 0) and *high* (*c* = *h*) and *low* (*c* = *l*) confidence for condition 3 for subject 5 (see the rightmost violin plot in the first row of Figure 1). We binarized the subject’s nearly continuous report of confidence at the optimal point shown by the magenta bar in the figure (i.e., a threshold of 0.72). In this case, the probability of being correct is *P*(*r* = 1) = 855/998, with an entropy of 𝓗_2_(*r*) = 0.593 bits; the probability of high and low confidence are *P*(*c* = *h*) = 420/998 and *P*(*c* = *l*) = 578/998 respectively; the conditional probability of being correct given *high* confidence is *P*(*r* = 1|*c* = *h*) = 415/420 with an entropy of *h*_2_[*P*(*r*|*c* = *h*)] = 0.093 bits; and the conditional probability of being correct given *low* confidence is *P*(*r* = 1|*c* = *l*) = 440/548 with an entropy of *h*_2_[*P*(*r*|*c* = *l*)] = 0.793 bits. Thus the full mutual information is

$$\begin{array}{l} \text{meta}–𝓘={𝓗}_{2}\left(r\right)-P\left(c=h\right)\times h_{2}\left[P\left(r|c=h\right)\right]-P\left(c=l\right)\times h_{2}\left[P\left(r|c=l\right)\right]\\ \;=0.593\;-\frac{420}{998}\times 0.093\;-\frac{578}{998}\times 0.793\\ \;=0.094 \end{array}$$

as in the figure.

**Table 1.**
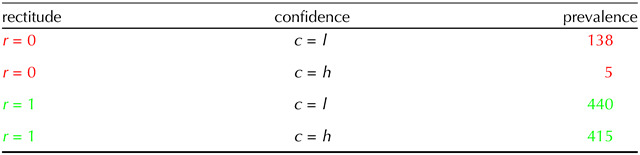
Calculation tableau for meta–𝓘 for condition 3 for subject 5 (see also Figure 1). Here, we see the prevalence of the four combinations of being correct (*r* = 1) or incorrect (*r* = 0) and having high (*c* = *h*) or low (*c* = *l*) confidence, dividing the subject’s judgement at the single threshold of 0.72 shown by the magenta line in Figure 1

Figure 2C generalizes this to show meta–𝓘 for two ways of turning the (nearly) continuous measure of confidence that the subjects reported into a set of mutually exclusive bins.^1^ The lower (magenta) border of the lozenge for each condition (distinguished by the fill colour) is the result of choosing the best binarization of confidence (something that Shekhar and Rahnev (2021) explored explicitly). The short horizontal magenta lines on the violin plots of Figure 1 show where this binary separation falls for those three subjects—trying to separate green and red masses vertically. These thresholds are ultimately an expression of the bias in confidence reporting of the subjects—we see their levels roughly reflecting how the subjects employ the confidence scale. The upper (green) border of each lozenge shows the case of three optimized levels of confidence arising from the two thresholds shown as lower and upper triangles in Figure 1. First, note that meta–𝓘 values are (by construction) higher for the extra bins—a phenomenon we explore further below. Second, the subjects are sorted as in Figure 2A (by meta–*d*′ in the lowest contrast condition); however the lozenge for this condition is almost monotonic; and the lozenges for the higher contrasts have a similar degree of monoticity to those in meta–*d*′, suggesting that meta–𝓘 is somewhat consistent with meta–*d*′. Along with this, we see that meta–𝓘 also increases with *d*′—although we will later qualify this finding—it arises here partly because of the rather modest levels of the actors’ *d*′s in this study (with most M-ratios being less than 1).

meta–𝓘 is a measure of metacognitive *sensitivity*. As for the relationship between meta–*d*′ and the M-ratio, measuring metacognitive *efficiency* requires normalizing for a quantification of potentially available information about confidence. Guggenmos (personal communication) thus suggested taking the analagous step of calculating a meta–𝓘-ratio by normalizing meta–𝓘. One possible normalizer would be a quantity we write as meta–𝓘(*d*′) that would arise as the value of meta–𝓘 for a first-order rater following a conventional signal detection theory analysis based on the actor’s *d*′ where

$$\alpha ∼𝒩\left(d,\frac{4}{{\left(d^{\prime }\right)}^{2}}\right)\;a=\mathrm{sign}\left(\alpha \right)\;c=\frac{1}{1+\exp\left(-d^{\prime }|\alpha \right)}$$

The green curve in Figure 2D shows meta–𝓘(*d*′) as a function of *d*′. For relatively low values of *d*′, as seen in Shekhar and Rahnev (2021), this increases with *d*′. However, for large *d*′, it decreases again, since meta–𝓘 is bounded above by the entropy of the accuracy 𝓗_2_(*r*)—and as *d*′ rises, the actor becomes increasingly accurate, and so this entropy decreases.

This manoeuvre of normalizing meta–𝓘 by meta–𝓘(*d*′) parallels the M-ratio’s use of *d*′ itself to normalize meta–*d*′. It captures the inability of a first order actor with poor perceptual abilities to judge confidence well; and the consequences for metacognitive sensitivity of the lack of variability in the rectitude of an actor with excellent perceptual abilities. We write meta–${𝓘}_{1}^{r}$ = meta–𝓘/meta–𝓘(*d*′), and show it in Figure 2E for the two and three confidence bins of Figure 2C, but now sorted by the M-ratio of the lowest contrast condition (i.e., the sort used in Figure 2B). As for the M-ratio, we see that this normalization has more nearly equated the estimates of meta-cognitive efficiency for the three conditions, to a roughly equivalent degree to the M-ratio.

Figure 2F shows the relationship between the M-ratio and meta–${𝓘}_{1}^{r}$ for all subjects and all conditions for the case of two (magenta) and three (green) confidence bins (with the cases joined by vertical lines). We can see that there is a very close relationship between the M-ratio and meta–${𝓘}_{1}^{r}$, at least in this regime of actors and raters, confirming the impression from comparing Figures 1B and 1D.

How, though, should we think about the fact that there are apparently different values of meta–𝓘 for different numbers of confidence bins? All else being equal, a rater that can accurately distinguish a larger number of levels of accuracy should reasonably be considered to be more metacognitively sensitive and efficient—since this rater can offer a finer perspective on the chance of failure. Equivalently, meta–𝓘 will benefit from the deblurring of the ratings that occurs when they are split into more levels, at least provided that these levels are used well. This is not a property of meta–*d*′ or the M-ratio—the main consequence of increasing the granularity of the confidence report is to affect the fitting process for estimating the rater’s equivalent *d*′—it has no direct bearing on that version of sensitivity or efficiency.

To assess the consequence of increasing granularity, we evaluated the average across the subjects in Shekhar and Rahnev (2021) of the ratio of meta–𝓘 for between two and ten confidence bins and for just two bins. Here, we approximately optimized the thresholds on a subject- and condition-specific basis (Figure 2G). From the increase with the number of bins, it is apparent that the subjects are able on average to report confidence at at relatively fine granularity—particularly in the most difficult (blue) contrast condition—but that this ultimately saturates (with many fewer than the ∼500 confidence bins of the experimental report). One wrinkle here is that we calculated the efficiency normalizer, meta–𝓘(*d*′), assuming continuous confidence judgements can be made by a first-order rater (i.e., with an infinite number of correctly-employed confidence bins). This is reasonable, because this estimate is based on a model that allows calculation to arbitrary accuracy. However, it could be questioned as a comparator, and it would also be possible to normalize by a version of meta–𝓘(*d*′, *b*) that uses to optimal effect the same number (*b*) of confidence bins as the empirical rater.

Figure 2H reports the result of normalizing meta–𝓘 by the theoretical upper bound 𝓗_2_(*r*) we mentioned above rather than meta–𝓘(*d*′). We call the resulting measure of metacognitive efficiency meta–${𝓘}_{2}^{r}$. 𝓗_2_(*r*) is shown as a function of *d*′ in the black curve in Figure 2D. This also accounts well for the fact that, for high *d*′ for the actor, metacognitive sensitivity cannot be high, since, as we noted, there is little entropy in 𝓗_2_(*r*) to reduce by 𝓗_2_(*r*|*c*). However, in cases such as the second order model we consider later in which the rater can have access to much better information than the actor, it allows us to assess the efficiency in absolute term. The data in Figure 2H suggest that this regime is not relevant for the data in Shekhar and Rahnev (2021), in that the raters appear to be generally rather worse than the actors.

A final issue with information theoretic measures concerns estimation of entropies and conditional entropies. The mutual information associated with continuous variables, such as confidence in some experiments, is known to be hard to estimate, because of biases, and so care is necessary (Kozachenko & Leonenko, 1987; Paninski, 2003; Panzeri & Treves, 1996; Witter & Houghton, 2021). Biases are typically weaker for discrete variables, which are employed in many experiments on confidence. Here, we use randomized or exact permutation methods as a simple way to correct for biases.

## A SECOND-ORDER DECISION-MAKER

**Figure 3.**
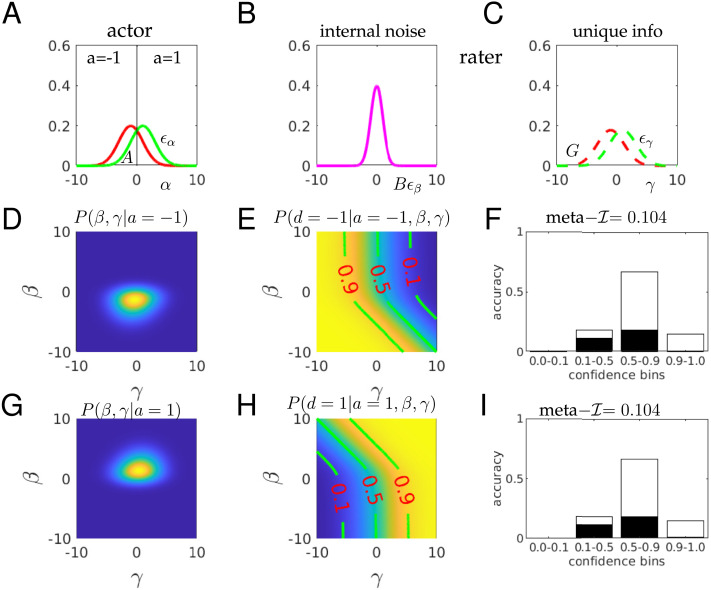
**Mutual information calculations for a realized second-order model.** (A) the actor observes a signal *α* = *d* + (2/*d*′)*ϵ*_*α*_ (red for L: *d* = −1; green for R: *d* = +1) and makes an unbiased decision *a* for *d* = ±1 at the boundary *α* = 0. (B, C) The rater receives two pieces of information: *β* = *α* + *Bϵ*_*β*_; *γ* = *d* + *Gϵ*_*γ*_ where all *ϵ* are standard 𝒩(0, 1) and independent. *γ* is called unique since it contains information about *d* that is not shared with the actor. Here *d*′ = 1; *B* = 1; *G* = $\sqrt{5}$. (D) The density *P*(*β*, *γ*|*a* = −1) slightly favours the lower left quadrant, but with substantial noise. The distribution integrates to 1; color scale not shown for convenience. (E) The conditional probability *P*(*d* = −1|*a* = −1, *β*, *γ*) is the accuracy afforded by the rater’s information set (*a* = −1, *β*, *γ*). If *β*, *γ* ≪ 0, then the decision *a* = −1 is likely to be true. The contour lines show the boundaries where this objective confidence crosses the values shown—the enclosed regions are where objective confidence ratings would be provided. (F) If we consider the regions of *β*, *γ* that define these bins of confidence, we can assess the expected accuracy—defined by the combination of the probability of ending up in one of the confidence bins *c*(*a* = −1, *β*, *γ*) and the chance of being correct (white) or incorrect (black) in that bin. The mutual information 𝓘(*r*, *c*) between being correct and *c* (given *a* = −1) is 0.104 bits. (G–I) The same as (D–F) except for the case that *a* = 1. Since the problem is symmetric, this is essentially the same as for *a* = −1.

In order to examine meta–𝓘 and meta–${𝓘}_{\left\{1,2\right\}}^{r}$ in more detail, we turned to a simulation which allows us to abstract the relevant factors away from the noise associated with the ratings of individuals. We simulate choices and ratings from a simple, realized form of a second-order decision-making (Fleming & Daw, 2017; Jang et al., 2012; Mamassian & de Gardelle, 2022). On any trial, the actor and rater collectively receive three Gaussian distributed signals that bear on a true underlying quantity *d* = ±1 (Figure 3A–C); the actor generates a binary choice *a* (Figure 3A); and the rater generates one of a number of discrete confidence values *c* (Figure 3E and 3H). Here:

$$\alpha =d+A\epsilon_{\alpha }\;\text{is}\;\text{the}\;\text{primary}\;\text{decision}\;\text{variable}\;\text{with}\;\text{type}‐1\;\text{sensitivity}\;d^{\prime }=2/A$$ (5)

$$\beta =\alpha +B\epsilon_{\beta }\;\text{allows}\;\text{the}\;\text{rater}\;\text{partial}\;\text{insight}\;\text{into}\;\text{the}\;\text{basis}\;\text{of}\;\text{the}\;\text{actor}’\text{s}\;\text{decision}$$ (6)

$$\gamma =d+G\epsilon_{\gamma }\;\text{provides}\;\text{the}\;\text{rater}\;\text{with}\;\text{independent}\;\text{or}\;\text{unique}\;\text{information}\;\text{about}\;d$$ (7)

where {*ϵ*_*α*_, *ϵ*_*β*_, *ϵ*_*γ*_} are independent standard 𝒩(0, 1) distributed random variables. We assume that *d* = ±1 with equal probability, and that the actor is unbiased, and so makes a decision based on the sign of *α*, with *a* = −1 if *α* < 0, *a* = 1 if *α* > 0 and is indifferent if *α* = 0. The rater bases its choice on the observation of the action *a* and the random variables *β* and *γ*, whose combination arranges for potentially partial correlation between the actor’s and rater’s information about the true state of the stimulus, *d*, as in the standard second order model. Here, the rater’s confidence *c* = *P*(*a* = *d*|*a*, *β*, *γ*) is the probability that the actor’s action *a* was correct given all the information that the rater possesses.

Although for didactic convenience, the realization of the information structure relating actor and rater is different here from the canonical stochastic detection and retrieval model of Jang et al. (2012), the ultimate statistical relationships are the same; we provide a translation in the Supplement. Thus, briefly, and as discussed at length in Fleming and Daw (2017), this model has various natural limits of metacognitive interest. In the case that *B* → 0; *G* → ∞, the rater has exactly the same information as the actor, and so could act like the naive Bayesian decision theorist above. Then, meta–*d*′ would be the same as *d*′, and the M-ratio would be 1. In the opposite case, *B* → ∞; *G* → 0, the rater would have perfect knowledge about the rectitude of the actor’s choice (based on the equivalent of perfect post-decisional information, also known as a confidence ’boost’; Mamassian and de Gardelle (2022)) and so could be as sensitive as it is possible to be. If *B* → ∞; *G* → ∞, then the rater has no specific information about the basis of the actor’s choice on a trial, and so, like the incompetent weather forecaster, could do no better than reporting the overall expected accuracy on each trial (which here is Φ(*d*′/2), where Φ is the cumulative distribution function for a normal distribution). In non-limiting cases, *β* provides the rater with information about the data on which the actor based their choice, which she has to combine with her private, e.g., post-decisional, information about *d*.

It is didactically convenient to consider response-specific confidence, although here, the symmetry of the problem means that all the measures will be the same for *a* = −1 and *a* = 1. Figure 3D and 3E show the two critical quantities that govern confidence judgements for *a* = −1 (for the case that *d*′ = 1; *B* = 1, *G* = $\sqrt{5}$). First, Figure 3D shows the posterior density that the rater will observe *β* and *γ* given that *a* = −1. These values slightly favour the lower left quadrant (*β* < 0; *γ* < 0), since *a* = −1 implies that the actor saw *α* < 0. Note that this preference is stronger as a function of *β* than *γ*; this is because *B* is quite small, and we know that *α* < 0 (since *a* = −1). Second, Figure 3E shows the optimal confidence *P*(*d* = −1|*a* = −1, *β*, *γ*) that the rater would have about *d* = −1 given all the information in her possession. The plot shows contours at {0.1, 0.5, 0.9} to indicate more precisely the shape of this distribution. Coarsely, if *β* is very negative, which, because *B* is small, likely means that *α* was very negative, then the rater is rather confident that *a* = −1 is correct, unless *γ* is very large and positive, to counteract this. The slopes of the contours for negative *β* largely reflect the relative information about *d* in *α* and *γ*. If *β* is very positive, then since *a* = −1, it can only be that *α* is very close to 0, and so the rater has to rely on *γ*, implying that the contours run largely perpendicular to the *γ* axis.

Figure 3F shows the consequence for the confidence ratings. Here, we consider the four bins implied by the contours in Figure 3E): {[0, 0.1), [0.1, 0.5), [0.5, 0.9)[0.9, 1.0]}. These bins were chosen to keep them separated on the plot; we consider issues of the nature of the bins later. The total height of each bar integrates the total probability mass (from Figure 3D) that ends up in each of the regions delineated in Figure 3E. This quantifies the fraction of confidence reports that will end up in each confidence bin. For each of these confidence reports, the actor could be correct or incorrect; we show the expected proportion of correct reports in white; and incorrect reports in black. If the confidence bins were very narrow, then since all calculations are probabilistically correct, the relative heights of black and white parts of a bar would be given by just the confidence level associated with this bar (since this is exactly what the confidence quantifies). However, since the confidence regions are rather wide, we have to calculate a weighted average, where the weights are purely determined by the probability mass in Figure 3D and the quantity that is averaged is the precise confidence in Figure 3E. Thus, for instance, the mean accuracy in the [0.5, 0.9) bin is slightly less than the centre of this internal (0.75). In this instance, we can calculate meta–*d*′ = M-ratio = 1.7 based on the statistics in these confidence bins.

Figure 3G–H show exactly the same as Figure 3D–F, but for the case that *a* = 1 instead. The distributional plots are mirror symmetric, favouring positive rather than negative values of *β*, *γ*. The confidence values in Figure 3H are exactly the same as in Figure 3F, since this rater is just as good for *a* = 1 as for *a* = −1.

We now consider meta–𝓘 and meta–${𝓘}_{\left\{1,2\right\}}^{r}$ for this case. First, the actor is 69% accurate (with *d*′ = 1), making the unconditional entropy 𝓗_2_(*r*|*a* = −1) = 0.89 bits. Second, *P*(*c*|*a* = −1) is the total height of the bars in Figure 3F for confidence rating *c* ∈ {[0, 0.1), [0.1, 0.5), [0.5, 0.9)[0.9, 1.0]}, and *P*(*r*|*c*, *a* = −1) is the ratio between the black and white portions of those bars. The individual entropy terms for the bars in Figure 3F (defined by 𝓗_2_[*P*(*r*|*c* ∈ bin_*i*_, *a* = −1)]) are 0.42, 0.95, 0.84, 0.34 bits respectively for the four bins, making the total remaining uncertainty about the accuracy as 𝓗_2_(*r*|*c*, *a* = −1) = 0.79. This leaves meta–𝓘 = 0.89 − 0.79 = 0.1 bit, and, since meta–𝓘(*d*′ = 1) = 0.052 bits, we have meta–${𝓘}_{1}^{r}$ = 2. Here, although the rater is therefore more accurate than the actor (as similarly reflected by the M-ratio), her absolute efficiency meta–${𝓘}_{2}^{r}$ = 0.12 is rather low, since the rater’s unique information *γ* is subject to quite some noise, with *G* being large.

**Figure 4.**
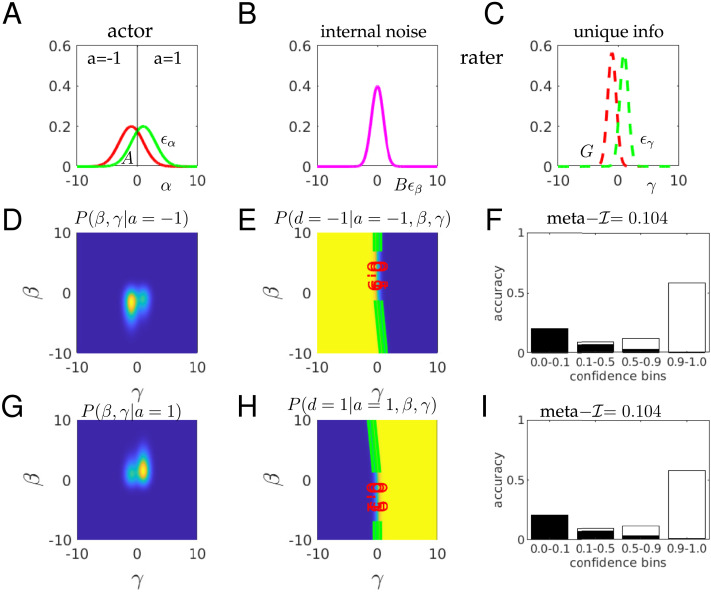
**Mutual information calculations for a realized second-order model.** This figure is the same as Figure 3, except that the standard deviation of the rater’s unique information *γ* is *G* = $\sqrt{0.5}$. The bimodal distributions in (D, G) come from the two narrow possibilities for *γ*, with the weight on *γ* being near to −1 being higher in (D), because *a* = −1 there. Now the confidence contours are defined almost exclusively by *γ* (the near vertical arrangements in E and H); and the accuracy bins are nearly exclusively one colour (when the rater’s confidence is 0–0.1, the actor is almost always incorrect.

Figure 4 shows the same as Figure 3, but for the case that the rater enjoys a much greater amount of unique, post-decisional, information (with *G* = $\sqrt{0.5}$; Figure 4C). Figure 4D now shows bimodality, since *γ* is only likely to be near *d* = ±1, and less likely to take a value near 0. The mode associated with *γ* = −1 has a greater mass than that for *γ* = 1, since *a* = −1. The conditional distribution in Figure 4E now shows a much starker contrast—with the highly accurate *γ* being the main determinant of the confidence in the actor’s choice (so if *γ* > 0, then the rater is rather confident that the actor erred). Figure 4F shows the consequence of this for the rating buckets. Now, the extreme values are much more likely—and are duly more pure in the sense that the rater can be rather sure about the rectitude of the actor. Here, meta–*d*′ = M-ratio = 4.5, showing the benefit of the well-informed rater. Again, the case for *a* = 1 (Figure 4G–H) is the mirror image of the case for *a* = −1.

If we carry out the same calculations of meta–𝓘 and meta–${𝓘}_{\left\{1,2\right\}}^{r}$ for this case, we observe that the individual entropy terms for the bars in Figure 4F are 0.15, 0.84, 0.82, 0.11 respectively. However, the fact that most of the weight in the average is on the outer two bars means that the total remaining uncertainty about the accuracy as 𝓘_2_(*r*|*c*, *a* = −1) = 0.27. This makes meta–𝓘 = 0.62 bits, and meta–${𝓘}_{1}^{r}$ = 12. In this regime, the M-ratio and meta–${𝓘}_{1}^{r}$ diverge. However, the actor’s performance is not a good yardstick for the rater, since the rater has substantially more information. Thus, meta–${𝓘}_{2}^{r}$ = 0.7 is a more useful measure of the high absolute efficiency of the rater, which reflects the high signal to noise ratio of the rater’s unique information, with *G* being small.

**Figure 5.**
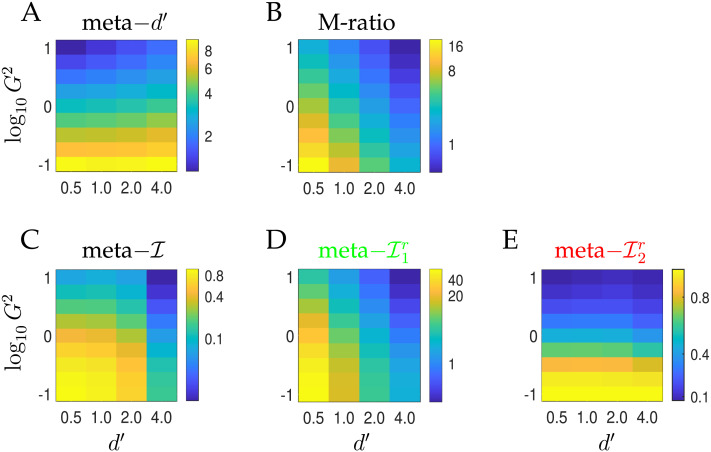
**Meta-cognitive sensitivities and efficiencies for the second order observer as a function of *d*′ and log_10_ *G*^2^ for the case that *B* = 1 and there are four confidence bins that are optimized to maximize meta–𝓘 (and so differ for each combination of *d*′ and *G*^2^).** (A, B) meta–*d*′ and the M-ratio, with the latter suggesting that the increase in meta–*d*′ for larger *d*′ is not a sign of efficiency. (C) meta–𝓘, showing that as *d*′ gets large, the mutual information decreases, since the entropy of accuracy, 𝓗_2_(*r*), decreases. (D) Normalizing meta–𝓘 by the mutual information meta–𝓘(*d*′) leads to values that are close to the M-ratio (B) away from the regime in which *G*^2^ is small so the rater has access to higher quality information than the actor. (E) Normalizing meta–𝓘 by the entropy of the accuracy 𝓗_2_(*r*) provides a measure of absolute efficiency which is roughly constant for small *G*^2^, as the rater’s unique information dominates.

Figure 5 compares the various metacognitive sensitivity and efficiency measures for various values of the actor’s type 1 sensitivity *d*′, and for different qualities of the unique information of the rater *G*^2^. Here, as in Figures 3 and 4, *B* = 1. By contrast with the earlier figures, however, the rater optimally deployed four confidence bins.

These plots cover the two regimes discussed earlier. In one, where *G*^2^ is not too small, the rater is at least co-dependent on the information that the actor used. Here the M-ratio (B) and meta–${𝓘}_{1}^{r}$ (D) largely agree (although, as we will see later, meta–${𝓘}_{1}^{r}$ correctly exploits extra confidence bins). However, in the other regime, where the rater is mostly dependent on its own source of information (*G*^2^ is small), and the actor’s performance is poor, then both the M-ratio and meta–${𝓘}_{1}^{r}$ diverge. Here, the absolute efficiency, meta–${𝓘}_{2}^{r}$ (E), of the rater is more relevant. Indeed, we can see that meta–${𝓘}_{2}^{r}$ is largely constant as a function of *d*′ for very low *G*^2^. This property is shared by meta–*d*′; however meta–*d*′ lacks an appropriate scale (since the actor’s *d*′ is not an appropriate baseline).

**Figure 6.**
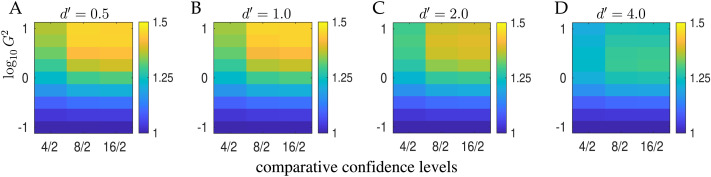
**The effect of the number of levels of confidence.** The ratio between meta–𝓘 (or equivalently meta–${𝓘}_{\left\{1,2\right\}}^{r}$ values) for 4, 8, 16 confidence bins to that for 2 confidence bins for *d*′ = 0.5 (A), *d*′ = 1 (B) and *d*′ = 2 (C) for 4, 8, or 16 levels of confidence (all optimized) and for values of *G*^2^ between 0.1 and 10. The scales are set to be the same for all three heatmaps. Here *B* = 1.

As we saw in Figure 2F and 2G, one particular issue that spurs the use of meta–𝓘 is the effect of increasing the granularity of the confidence rating. Figure 6 examines this issue from the perspective of our second-order rater, showing how meta–𝓘 (the same would be true of the efficiency measures) increases with the number of confidence levels for different qualities of actor and different amounts of independent information provided to the rater (quantified as by *G*^2^). Here, the thresholds defining the levels were again set to optimize meta–𝓘. In this idealized case, the extra levels are never harmful, but the degree to which they are helpful varies quite substantially. As we saw in Figure 2G, the increase is greater for lower *d*′ (condition 1 in that figure); it also grows with *G*^2^. Various factors are involved: for instance whether there is such certainty of the actor (*d*′ = 4) or the rater (*G*^2^ ≃ 0.1) that two levels suffice. Note that these plots show the benefit as the ratios between 4, 8, 16 levels and 2 levels; as expected the total meta–𝓘 decreases as *G*^2^ gets larger.

Even for a given number of levels, the mutual information can vary as a function of the actual confidence intervals. For instance, for the cases of Figures 3 and 4, if we use four evenly spaced levels (*c* ∈ {[0, 0.25), [0.25, 0.5), [0.5, 0.75), [0.75, 1.0]}) rather than the uneven ones (*c* ∈ {[0, 0.1), [0.1, 0.5), [0.5, 0.9)[0.9, 1.0]}) in the figures, meta–𝓘 increases to 0.12 bits for the case of Figure 3, and decreases to 0.60 bits for the case of Figure 4.

**Figure 7.**
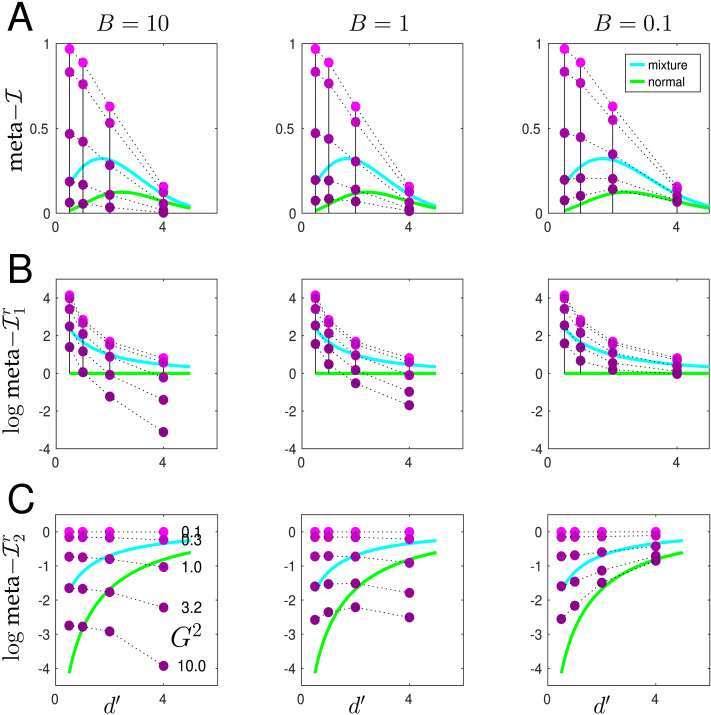
**Hypo and hyper-sensitivity.** The continuous lines show meta–𝓘 (A), log meta–${𝓘}_{1}^{r}$ (B) and log meta–${𝓘}_{2}^{r}$ (C) for the naive first order Bayesian case with continuous confidence levels across different values of *d*′ for the case of standard signal detection theory (green; discriminating two Gaussian distributions with means *d* = ±1 and standard deviation *σ* = 2/*d*′) and for the extreme mixture case (cyan; with an actor that is 50% correct with probability 2(1 − Φ(*d*′/2)) and 100% correct otherwise). The magenta points are from the second order model with the three values of *B* shown in the titles and the values of *G*^2^ = {10, 3.7, 1, 0.27, 0.1}, from bottom to (top) for the case of 16, optimally-spaced confidence levels. Points for the same value of *G* are connected by dotted lines for graphical convenience.

The efficiency measures meta–${𝓘}_{1}^{r}$ and meta–${𝓘}_{2}^{r}$ are ways of measuring the hypo- and hyper-metacognitive sensitivity for meta–𝓘. In Figure 7, we compare meta–𝓘 (A) and the efficiency ratios (B, C) for particular idealized actors and raters (green and cyan lines) with the same measures for the actual second order rater of the previous figures for the maximum number (16) of optimized confidence bins (magenta points). The green line comes from the same rater that defined meta–𝓘(*d*′), i.e., the case of standard signal detection theory in which the actor discriminates two Gaussian distributions with means *d* = ±1 and common standard deviation *σ* = 2/*d*′, and the rater acts as a naive Bayesian type 1 rater with a continuous range of confidence levels. Thus, the green lines in Figure 7A are the same as in Figure 2D; and the green lines in Figure 7B are flat at 0, since meta–${𝓘}_{1}^{r}$ is defined as the ratio between meta–𝓘 for a rater and meta–𝓘(*d*′) itself. The absolute efficiency of this idealized rater decreases as *d*′ gets smaller, since the actor makes more errors, but the rater lacks the information to discriminate them.

The cyan lines show meta–𝓘 and meta–${𝓘}_{\left\{1,2\right\}}^{r}$ for the less standard model of actor and type 1 rater that was considered as a *reductio ad absurdum* by Rahnev and Fleming (2019), for which the error rate associated with a conventional *d*′ (which is *p*_err_ = 1 − Φ(*d*′/2)) comes from a mixture model in which the actor and rater are either completely guessing (with actual and believed probability of error 50%); happening with a mixture proportion of 2*p*_err_; and actual and believed probability of error 0%, with a mixture proportion of 1 − 2*p*_err_. The values of meta–𝓘 and meta–${𝓘}_{\left\{1,2\right\}}^{r}$ for this rater are higher than for the standard one, since there is extra information about the source of errors. This reminds us that *d*′ is an incomplete measure of the actor’s process, again making it important to interpret cautiously measures such as the M-ratio and meta–${𝓘}_{1}^{r}$ that use it directly for normalization.

One could consider meta–𝓘 and meta–${𝓘}_{\left\{1,2\right\}}^{r}$ values lower than these numbers to be hypo-efficient; and ones larger than these to be hyper-efficient—at least relative to these raters. The magenta points show that as *G*^2^ decreases (bottom to top), the second order model generally goes from hypo to hyper-efficiency; but *B* also plays a role. This is clearest for the meta–${𝓘}_{1}^{r}$, where for intermediate values of *G*, the rater becomes hypo-efficient as *d*′ grows for large *B*, when the rater cannot prosper from the extra information the actor enjoys.

## DISCUSSION

Fleming and Lau (2014) noted that standard ways of quantifying metacognition are based on distributions such as those in Figures 1 and 3F, 3H (at least if extra factors such as the time participants take to rate confidence are not taken into account; Desender et al., 2022). Indeed, Nelson (1984) listed eight such measures, which do not include meta–*d*′ or meta–𝓘 or meta–${𝓘}_{\left\{1,2\right\}}^{r}$, being measures of metacognitive sensitivity and efficiency that we advocate here. meta–𝓘 is the mutual information between the actual accuracy of the choices of the actor and the confidence ratings produced by the rater about those choices. This is a simple function of the same statistics used to calculate meta–*d*′ and the M-ratio, and shares some of the desirable properties of those quantities (along, of course, with the less desirable ones rooted in assumptions such as that the decision-making process is stationary, with a fixed strength of evidence; Rahnev & Fleming, 2019). However, like correlation measures (Nelson, 1984), meta–𝓘 has the additional benefit of not depending on a potentially imperfect fit to a model of type 1 choice that might not be exactly appropriate, and it also scales appropriately with such factors as the number of levels of confidence. Note that Bowman et al. (2022) suggested using the mutual information to measure a form of type 1 sensitivity in a study of awareness rather than confidence.

That apparent metacognitive efficiency can increase with the number of levels with which subjects rate confidence suggests that in experiments collecting confidence ratings, it would be worth paying some extra attention to the way that reports are solicited. In the data from Shekhar and Rahnev (2021), metacognitive efficiency increased up to 10 levels of reporting—at least on average—something we could observe clearly by looking at alternative quantizations of the nearly continuous confidence data they had collected. It would be interesting to carry out this exercise on other data. It should be noted that evaluating mutual information measures for a truly continuous confidence report is tricky from modest amounts of data, because of known biases (Kozachenko & Leonenko, 1987; Paninski, 2003, Panzeri & Treves, 1996; Witter & Houghton, 2021); and so further study in particular cases would be most valuable.

We illustrated meta–𝓘 and meta–${𝓘}_{\left\{1,2\right\}}^{r}$, and compared them with the other measures, using both data from Shekhar and Rahnev (2021) and a simple case of a second order rater (Fleming & Daw, 2017), which is not restricted to having confidence at least as large as 50% (as would be true of a naive type 1 Bayesian), and can be either hypo- or hyper-metacognitively efficient.

The evaluation of the mutual information is completely bias-free. Of course, as noted in the Introduction, bias can affect the mutual information, by affecting the utilitization of the confidence levels, thereby increasing the conditional entropy of the accuracy given the rating (𝓗(*r*|*c*, *a*)). However, like all information theoretic quantities (though unlike the M-ratio; Xue et al., 2021), meta–𝓘 is completely unaffected by the *labels* that are given to the confidence levels—it is only influenced by the conditional accuracy that these levels afford. Indeed, meta–𝓘 would be unaffected if the labels were scrambled, so that subjects notionally reported ‘high confidence’ in the actor’s choice to mean that an error was likely, and ‘low confidence’ when an error was not. This is also unlike meta–*d*′, which makes assumptions about the appropriate monotonicity of the reporting levels in order to be able to calculate a notional type 1 *d*′. It might be possible to include in the optimization process that leads to the evaluation of meta–*d*′ an additional reordering of the confidence levels—although this would then create a complex combinatorial optimization problem (with *n*! possible orders for *n* levels). It would be interesting to examine other information theoretic mechanisms that might preserve at least the order of the levels. For instance, one might imagine the act of reporting as being like a noisy channel, in which subjects can stochastically report levels that are somewhat different from their true confidence. A treatment that would have this effect (albeit, technically, by varying the confidence criteria rather than the confidence signal) is exactly what was suggested by Shekhar and Rahnev (2021) as a process model for metacognitive inefficiency in the data that we analyzed above (see also Guggenmos, 2022). One limit of such noisy processes might offer a good formalization of the common empirical practice of recording confidence on a continuous scale (e.g., using a slider), but then for the experimenter to create a set of bins whose width would be determined by the structure of the stochastic report.

We noted that different positioning of the bins of confidence (even keeping the number constant) could lead to different values of meta–𝓘. We approximately optimized the bins on a case by case basis,^2^ a bit like efficient coding of sensory information (Laughlin, 1981; Zhaoping, 2006), where coding levels are adjusted to reflect the ‘natural’ statistics of the information that is being coded. In the context of social inference, Bang et al. (2017) suggested maximising the entropy of confidence reports. The mutual information of Equation 1 can equivalently be written as the difference between the unconditional entropy of the confidence reports (given the action) and the conditional entropy given the accuracy (and the action). Thus maximizing the entropy can be beneficial for improving meta–𝓘. However, the consequences for the second term of the mutual information, namely the conditional entropy of the confidence reports given the accuracy (and the action), also need to be taken into account. It would certainly be interesting to examine the optimal meta–𝓘 solutions in more detail. If, as in Bang et al. (2017), the confidence regions change over time (for instance, to optimize their utilization), then metacognitive effectiveness and meta–𝓘 will change too, and would need to be tracked in an appropriately dynamic manner, something that poses potential statistical problems. It has been noted that the test-retest reliability of the M-ratio is compromised when the number of rating levels increases, suggesting that it would be important to investigate the equivalent for meta–𝓘 and meta–${𝓘}_{\left\{1,2\right\}}^{r}$.

There is much discussion of the need for meta–*d*′ to be corrected by the type 1 sensitivity *d*′ in order to assess metacognitive efficiency—since cases with high *d*′ are intrinsically easier. This has some undesirable consequences—for instance if *d*′ is very low, then even a modest meta–*d*′ can lead to an extremely large M-ratio, something that has inspired the use of the difference (meta–*d*′ − *d*′) rather than the M-ratio, or the logarithm of the M-ratio in such circumstances. We showed that meta–${𝓘}_{1}^{r}$ has a similar issue and suggested that in the regime for the second-order model in which the rater is replete with its own sources of information that exceed those of the actor, it is more appropriate to consider the absolute efficiency of metacognition, normalizing meta–𝓘 instead by the unconditional entropy of the accuracy 𝓗_2_(*r*), which is an upper bound to the mutual information, and is the total available variability that confidence could potentially rate. This alternative ratio meta–${𝓘}_{2}^{r}$ assesses just how little of the available unconditional entropy of the accuracy is lost to a high conditional entropy (𝓗_2_(*r*|*c*, *a* = −1)) in the mutual information equation.

One might look at both meta–𝓘 or the meta–𝓘-ratio as potential correlates of brain structure and function (Baird et al., 2015; Fleming & Dolan, 2012; Fleming et al., 2010). Note that the informational quantities can formally also accommodate tasks which use multiple *d*′ values (for instance, if the quality of sensory information is different from trial to trial, as in the mixture curve of Figure 7). However, interpretative care is necessary (Rahnev & Fleming, 2019).

Like other information theoretic proposals in neuroscience, meta–𝓘 arguably offers more insight into bounds on the nature and quality of the computations involved in metacognition than into the neural realization of these computations. Process models such as those in Desender et al. (2022), Guggenmos (2022), and Shekhar and Rahnev (2021) or even the simple second order model that we considered (Fleming & Daw, 2017; Jang et al., 2012) are an attractive alternative, albeit adopting far stronger assumptions. Nevertheless, meta–𝓘 or meta–${𝓘}_{\left\{1,2\right\}}^{r}$ would be drop-in replacements for other measures such as the M-ratio for such assessments as volume-based morphometry for regions whose size is correlated with the quality of metacognition (Fleming et al., 2010). Of course, despite the attractive theoretical properties of information theoretic measures, they are far from unique in measuring the quality of raters. Indeed, so-called skill scores (a term of art in assessing the sort of probabilistic forecasters with which metacognition is concerned) can be based on (strictly) proper scoring rules (Gneiting & Raftery, 2007) (a class including the famous quadratic Brier score; Brier (1950); and the logarithmic scoring rule that underpins meta–𝓘). Correcting the evaluation of forecasters to reflect the difficulty of their forecasting tasks is also a concern in that literature.

In sum, the problem of confidence is inherently one of information—that the actor has about the true state of the stimulus; and that the rater has about the same quantity and about what the actor used. It therefore seems appropriate to use the methods of information theory to judge the relationship between the stimulus, the actor and the rater.

## ACKNOWLEDGMENTS

I am very grateful to Dan Bang, Stephen Fleming, Liam Paninski and Lion Schulz for most helpful discussions and comments on earlier versions of the manuscript, and particularly to Matthias Guggenmos for an extensive discourse and him and Li Zhaoping for suggestions about normalization of meta–𝓘. Funding was from the Max Planck Society and the Humboldt Foundation. PD is a member of the Machine Learning Cluster of Excellence, EXC number 2064/1—Project number 39072764 and of the Else Kröner Medical Scientist Kolleg “ClinbrAIn: Artificial Intelligence for Clinical Brain Research.”

## DATA AVAILABILITY STATEMENT

This study reused data that had been made publicly available based on the study of Shekhar and Rahnev (2021).

Data and code for reproducing the figures are available at https://github.com/Peter-Dayan-TN/metainf.git.

## Notes

^1^ See Shekhar and Rahnev (2021) for their analysis of the quantization of the continuous confidence report. They also performed a sophisticated examination of different models of metacognitive rating based on this (notably suggesting the influence of a particular sort of noise). However, for our present purpose of analyzing our model-free construct meta–𝓘, we will make a simple comparison with a binarized estimate of meta–*d*′.^2^ Although, in future work, it would also be worth using cross validation methods to estimate thresholds for discretizing confidence judgments.
