# Supplementary material for: Metacognitive Information Theory
Source: Open Mind (Camb). 2023 Jul 21;7:392–411. doi: 10.1162/opmi_a_00091 (PMC10449404; doi:10.1162/opmi_a_00091)
Supplement: Supplementary file 1 [file opmi-07-392-s001.pdf]

Supplement

Relationship with Fleming and Daw (2017)

Here, in order to show the effects of the separate components of the second order model from (Fleming and Daw, 2017, Jang et al., 2012), we modeled the rater as being provided with a noisy version β of the actor's signal (equation 6) as well as a noisy version of the true signal (equation 7).

In the original second order model, the signals that the actor and rater saw came from a bivariate (Gaussian) model, with

$$\begin{pmatrix} X_{\text{act}} \\ X_{\text{conf}} \end{pmatrix} \sim \mathcal{G}\left(\begin{pmatrix} d \\ d \end{pmatrix}, \begin{bmatrix} \sigma^2_{\text{act}} & \rho\sigma_{\text{act}}\sigma_{\text{conf}} \\ \rho\sigma_{\text{act}}\sigma_{\text{conf}} & \sigma^2_{\text{conf}} \end{bmatrix}\right) \tag{8}$$

Putting this into our terms, writing

$$\phi = \frac{G^2}{4/(d')^2 + B^2 + G^2}$$

we have the translation:

X_{act}	α
X_{conf}	$\phi\beta + (1-\phi)\gamma$
σ^2_{act}	$4/(d')^2$
σ^2_{conf}	$(1-\phi)G^2$
ρ	$\frac{2/d'}{\sqrt{4/(d')^2+B^2}} \frac{G}{\sqrt{4/(d')^2+B^2+G^2}}$
